# Incidence and Predictors of Thrombotic Complications in 4742 Patients with COVID-19 or Other Acute Infectious Respiratory Diseases: A Propensity Score-Matched Study

**DOI:** 10.3390/jcm10214973

**Published:** 2021-10-26

**Authors:** Antonio De Vita, Giuseppe De Matteis, Alessia d’Aiello, Salvatore Emanuele Ravenna, Giovanna Liuzzo, Gaetano Antonio Lanza, Massimo Massetti, Filippo Crea, Antonio Gasbarrini, Francesco Franceschi, Marcello Covino

**Affiliations:** 1 Department of Cardiovascular Sciences, Fondazione Policlinico Universitario A. Gemelli IRCCS, Università Cattolica del Sacro Cuore, 00168 Roma, Italy; alessia.daiello@gmail.com (A.d.); e91.rav@gmail.com (S.E.R.); giovanna.liuzzo@policlinicogemelli.it (G.L.); gaetanoantonio.lanza@unicatt.it (G.A.L.); massimo.massetti@unicatt.it (M.M.); filippo.crea@unicatt.it (F.C.); 2 Department of Clinical and Surgical Sciences, Fondazione Policlinico Universitario A. Gemelli IRCCS, Università Cattolica del Sacro Cuore, 00168 Roma, Italy; dr.giuseppedematteis@gmail.com (G.D.M.); antonio.gasbarrini@unicatt.it (A.G.); 3 Department of Emergency Medicine, Fondazione Policlinico Universitario A. Gemelli IRCCS, Università Cattolica del Sacro Cuore, 00168 Roma, Italy; francesco.franceschi@unicatt.it (F.F.); macovino@gmail.com (M.C.)

**Keywords:** acute infectious respiratory disease, COVID-19, SARS-CoV-2 infection, thrombosis, thromboembolic events

## Abstract

Background. A prothrombotic state, attributable to excessive inflammation, cytokine storm, hypoxia, and immobilization, is a feature of SARS-CoV-2 infection. Up to 30% of patients with severe COVID-19 remain at high risk of thromboembolic events despite anticoagulant administration, with adverse impact on in-hospital prognosis. Methods. We retrospectively studied 4742 patients with acute infectious respiratory disease (AIRD); 2579 were diagnosed to have COVID-19 and treated with heparin, whereas 2163 had other causes of AIRD. We compared the incidence and predictors of total, arterial, and venous thrombosis, both in the whole population and in a propensity score-matched subpopulation of 3036 patients (1518 in each group). Results. 271 thrombotic events occurred in the whole population: 121 (4.7%) in the COVID-19 group and 150 (6.9%) in the no-COVID-19 group (*p* < 0.001). No differences in the incidence of total (*p* = 0.11), arterial (*p* = 0.26), and venous (*p* = 0.38) thrombosis were found between the two groups after adjustment for confounding clinical variables and in the propensity score-matched subpopulation. Likewise, there were no significant differences in bleeding rates between the two groups. Clinical predictors of arterial thrombosis included age (*p* = 0.006), diabetes mellitus (*p* = 0.034), peripheral artery disease (*p* < 0.001), and previous stroke (*p* < 0.001), whereas history of solid cancer (*p* < 0.001) and previous deep vein thrombosis (*p* = 0.007) were associated with higher incidence of venous thrombosis. Conclusions. Hospitalized patients with COVID-19 treated with heparin do not seem to show significant differences in the cumulative incidence of thromboembolic events as well as in the incidence of arterial and venous thrombosis separately, compared with AIRD patients with different etiological diagnosis.

## 1. Introduction

SARS-CoV-2 is a novel coronavirus that is causing a pandemic outbreak of respiratory disease from the end of 2019 (COVID-19) [1,2]. Bilateral interstitial pneumonia is the most typical manifestation of COVID-19 and can be complicated by acute respiratory distress syndrome, multiorgan failure, and death in up to 30% of high-risk patients, requiring single or multiple organ support and admission in intensive care unit (ICU) [3,4,5,6]. A few studies have suggested that a prothrombotic state, attributable to excessive inflammation, cytokine storm, hypoxia, and prolonged immobilization, is a feature of severe COVID-19 infection and may predispose to both venous and arterial thromboembolic events, with adverse impact on in-hospital prognosis [7,8,9,10,11]. Postmortem examinations have identified both micro- and macro-embolism in COVID-19 casualties [12,13,14], though extensive thrombosis involving both pulmonary and extra-pulmonary microcirculation has rarely been described in small cohorts of hospitalized COVID-19 patients [15,16]. However, the exact prevalence of arterial and venous thrombosis in hospitalized COVID-19 patients remains uncertain and limited evidence exists to guide the prophylactic antithrombotic regimen [17,18,19,20].

Based on recent data, international guidelines recommend that hospitalized patients with COVID-19 and respiratory failure or severe comorbidities, as well as those requiring prolonged immobilization and intensive care, should receive pharmacological prophylaxis against venous thromboembolism (VTE), in the absence of contraindications [21,22,23].

Nevertheless, some studies have raised concern that, despite anticoagulant administration, up to 30% of patients with severe COVID-19 remain at high risk of thromboembolic events [24,25], with an incidence of thrombotic complications remarkably higher compared with that of other patient categories.

This study was aimed to (1) evaluate whether hospitalized patients with a diagnosis of COVID-19 present a different rate of arterial and venous thrombotic events, despite anticoagulant administration, compared to patients with other causes of acute infectious respiratory disease (AIRD); and to (2) identify the clinical predictors of thromboembolic complications in COVID-19 patients as well as in AIRD caused by other pathogens.

## 2. Materials and Methods

### 2.1. Study Design

This is a retrospective observational cross-sectional study enrolling patients with age ≥ 18 years admitted to our urban teaching hospital (with an emergency department treating >75,000 patients per year) because of confirmed AIRD, between 1 March 2020 and 1 March 2021. The diagnosis of AIRD was based on signs and symptoms (e.g., fever, dry or productive cough, dyspnea, tachypnea, hemoptysis) associated with either laboratory, radiological, or both signs of lower respiratory tract involvement [26].

Patients were divided into 2 groups based on the etiological diagnosis of the AIRD: (1) COVID-19 group and (2) no-COVID-19 group, which included all patients with any other infectious cause of the AIRD.

COVID-19 testing was based on the protocol released by the World Health Organization (WHO) [27]. Nasopharyngeal swab specimens were collected in all patients and SARS-CoV-2 RNA was detected by reverse-transcription polymerase chain reaction (RT-PCR). Chest X-ray and, when indicated, thoracic computerized tomography (CT) scan was performed to confirm the diagnosis.

### 2.2. Study Variables

The demographic characteristics (age and sex) and clinical data on admission were acquired from our institutional database. A history of known ischemic heart disease including any evidence of coronary artery disease (either previous myocardial infarction, coronary revascularization, documented obstructive coronary stenosis at angiography, or in combination), heart failure, peripheral artery disease (PAD), venous thromboembolism, or cerebrovascular disease (including previous stroke or transient ischemic attack), was reported as documented in patients’ clinical reports. The presence of pre-existing comorbidities, including chronic obstructive pulmonary disease (COPD) (Gold stage 3–4), severe renal failure (stage 4–5), chronic liver disease, cognitive impairment (as assessed by mini-mental state examination score ≤ 24 points), HIV infection, platelet disorder (including thrombocytopenia and thrombocytosis), connective tissue disease, solid tumor, and lymphoma/leukemia was also recorded.

The Charlson Comorbidity Index (CCI) was also reported for all patients as a score to assess the comorbidity level of patients by taking into account both the number and severity of 19 predefined comorbid conditions. Pharmacological therapy, with regard to the antithrombotic regimen, was also recorded on admission.

According to our internal institutional guidelines, hospitalized patients with a confirmed diagnosis of SARS-CoV-2 infection who reported either severe respiratory failure (arterial-to-inspired oxygen (PaO2/FIO2) ratio < 300, peripheral capillary oxygen saturation (SpO2) ≤ 93%, or both), as well as asymptomatic patients with a PADUA prediction score ≥ 4 [28], received a therapeutic dose of subcutaneous low molecular weight heparin (LMWH) (enoxaparin 1 mg/kg bid with dose adjustment according to age and creatinine clearance), whereas asymptomatic patients with a PADUA prediction score < 4 received prophylactic antithrombotic regimen (enoxaparin 40 mg/die) [29]. On the other side, patients with AIRD caused by other pathogens did not receive antithrombotic therapy except for a prophylactic dose of LMWH when considered at high risk of VTE according to clinical judgment.

Patients were followed until hospital discharge or death.

### 2.3. Ethical Approval

The study was conducted in accordance with the Declaration of Helsinki and its later amendments and was approved by the local Institutional Review Board (IRB #001705520). All patients admitted to the emergency department signed a comprehensive ethical agreement for the collection of blood samples and clinical data, for biobank and research purposes. 

### 2.4. Outcome Measures

The primary endpoint was the occurrence of any thrombotic/ischemic event in hospitalized patients with COVID-19 or other AIRD and consequently the effect of anticoagulant treatment administered to COVID-19 patients.

Arterial thrombosis was defined as the occurrence of acute myocardial infarction (AMI), acute ischemic stroke or transient ischemic attack (TIA), and acute peripheral artery thrombosis/embolism with critical limb ischemia, whereas venous thrombosis was defined as the occurrence of deep vein thrombosis (DVT), pulmonary embolism (PE), and other unusual sites of VTE (cerebral or splanchnic venous thrombosis).

Safety endpoints included the incidence of major, clinically relevant non-major, and minor bleeding events during the observation period, based on the International Society of Thrombosis and Hemostasis (ISTH) criteria [30,31].

### 2.5. Statistical Analysis

Data are reported as mean and standard deviation for continuous variables and number and proportions for discrete variables. Continuous variables were compared by analysis of variance, whereas proportions were compared by Fisher exact test or chi-squared test, as indicated. Differences between the 2 groups were adjusted for clinical variables that showed a significant or borderline (*p* ≤ 0.1) statistical difference, using multivariable logistic regression for discrete variables and a generalized linear model for continuous variables. Logistic regression was performed to identify independent predictors for total, arterial and venous thromboembolic events in the two groups. Only variables with a *p*-value ≤ 0.1 at univariable analysis were included in the multivariable models. A two-sided *p* < 0.05 was always required for statistical significance. Data were analyzed using SPSS 21.0 statistical software (SPSS Italia, Inc., Florence, Italy).

### 2.6. Propensity Score Matched Analysis

Since there were several major differences between the COVID-19 and no-COVID-19 groups in basal clinical variables, further analysis of differences in the rate of thrombotic events between the 2 groups was undertaken in a sub-population of COVID-19 and no-COVID-19 patients matched according to a propensity score. The independent variables in the propensity score model included age, gender, hypertension, diabetes mellitus, known ischemic heart disease, heart failure, PAD, previous TIA/stroke, previous DVT, chronic kidney disease, COPD, and history of solid tumor. Patients were matched by propensity score at a one-to-one ratio using the nearest-neighbor approach with no replacement and a caliper size of 0.1. A matched dataset containing 3036 patients (1518 in each group) was created. The standardized mean difference (SMD) for each covariate was calculated to evaluate the balance in baseline characteristics before and after matching. In addition, to further assess balance, the variance ratios of the continuous covariates in the COVID-19 and no-COVID-19 groups were calculated. Variables with an SMD ≤ 0.1 and variance ratio ≤ 1 were considered to be well-balanced between the two groups.

## 3. Results

Overall, 4742 patients, referred to our emergency department because of an AIRD, were considered for the study. The diagnostic workout confirmed the diagnosis of COVID-19 in 2579 patients (54.4%), whereas the other 2163 patients (45.6%) were diagnosed to have other causes of AIRD.

The main clinical characteristics of the two groups of patients are summarized in Table 1. COVID-19 patients were younger (*p* < 0.001) and included a higher proportion of male (*p* = 0.005) and hypertensive (*p* < 0.001) subjects compared to no-COVID-19 patients. However, a history of ischemic heart disease (*p* = 0.040), heart failure (*p* < 0.001), PAD (*p* < 0.001), previous DVT (*p* < 0.001), and previous stroke or TIA (*p* < 0.001) were significantly more frequent in the no-COVID-19 group. Similarly, COPD (*p* < 0.001), chronic kidney disease (*p* = 0.016), cognitive impairment (*p* < 0.001), connective tissue disease (*p* = 0.001), solid tumor (*p* < 0.001), and HIV infection (*p* = 0.008) were more frequent in the no-COVID-19 group compared with COVID-19 patients. 

On admission, patients of the no-COVID-19 group showed higher use of low dose aspirin (*p* < 0.001), P2Y12 inhibitors (*p* = 0.001), direct oral anticoagulants (*p* = 0.003) and statin (*p* < 0.001), whereas heparin use was more frequent among COVID-19 patients (*p* < 0.001).

### 3.1. Thrombotic Complications in COVID-19 and No-COVID-19 Group

The median duration of the observation per patient was 11 days (IQR 7–19). The rate of thrombotic complication of the two groups of patients is summarized in Table 2. As shown, during the hospitalization period, 271 thrombotic events (5.7%) occurred in the whole population of AIRD patients, 121 (4.7%) in the COVID-19 group and 150 (6.9%) in the no-COVID-19 group (*p* < 0.001). However, there were no differences in the rate of all kinds of thrombotic events after adjustment for the main clinical variables. 

The cumulative rate of arterial thrombosis was lower in COVID-19 compared to no-COVID-19 patients (2.4% vs. 3.7%, *p* = 0.016). No differences were found in the occurrence of AMI (*p* = 0.63), whereas a lower rate of ischemic stroke (*p* = 0.038) and peripheral artery thrombosis (*p* = 0.008) were reported in COVID-19 compared to the no-COVID-19 group. These differences, however, lost statistical significance after adjustment for the main clinical variables (Table 2). 

There was a tendency to lower cumulative VTEs in COVID-19 patients, although not achieving statistical significance (2.4% vs. 3.3%, *p* = 0.063). A higher rate of DVT was found in the no-COVID-19 group (0.9 vs. 0.2%, *p* = 0.002), whereas the rate of PE and cerebral or splanchnic venous thrombosis was not significantly different between the two groups (Table 2). The difference in the occurrence of DVT remained significant after adjustment for clinical variables (*p* = 0.028).

### 3.2. Propensity Score Matched Analysis

On the whole, 1518 COVID-19 and 1518 no-COVID-19 patients could be matched for pairwise comparison. The main clinical characteristics of these two groups are summarized in Table 3. As shown, the two groups were well matched for basal clinical features. 

No significant differences were detected in the incidence of arterial and venous thrombotic events between the two groups (Table 4). Any thrombotic event, in particular, occurred in 4.9% vs. 5.8% of COVID-19 and no-COVID-19 patients, respectively (*p* = 0.29).

**Figure 1:**
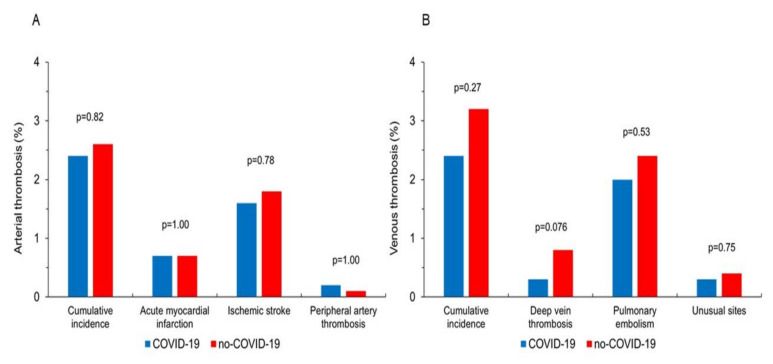
The graph shows the incidence of arterial (**A**) and venous (**B**) thrombotic events in the propensity score-matched subgroups of COVID-19 and no-COVID-19 patients.

The cumulative rate of arterial thrombosis (*p* = 0.82), as well as the incidence of AMI (*p* = 1.00), acute ischemic stroke (*p* = 0.78), and peripheral artery thrombosis (*p* = 1.00) was similar between the two groups of patients (Figure 1). Similarly, there were no differences between COVID-19 and no-COVID-19 patients in the cumulative rate of venous thrombosis (*p* = 0.27), PE (*p* = 0.53), and unusual sites of VTE (*p* = 0.75). The rate of DVT was slightly lower in COVID-19 patients compared to other AIRD patients (0.3% vs. 0.8%), although the difference failed to achieve statistical significance (*p* = 0.076) (Figure 1). 

### 3.3. Safety Endpoints

Major bleeding was reported in 32 (1.2%) COVID-19 and 18 (0.8%) no-COVID-19 patients (*p* = 0.20). Similarly, no significant differences were found in the occurrence of clinically relevant non-major bleeding (0.5% vs. 0.7%, *p* = 0.46) between the two groups of patients. Finally, minor bleeding was observed in 9 (0.3%) COVID-19 and 7 (0.3%) no-COVID-19 patients (*p* = 1.00).

### 3.4. Clinical Predictors of Thrombotic Events in AIRD Patients

#### 3.4.1. Cumulative Arterial and Venous Thrombosis

At univariate analysis, many clinical characteristics were significantly associated with arterial and venous thrombosis, respectively (Table 5 and Table 6). However, clinical variables independently associated with increased risk of arterial thrombosis at multivariable analysis only included age (OR 1.02, 95% C.I. 1.01–1.03; *p* = 0.009), diabetes mellitus (OR 1.50, 95% C.I. 1.03–2.19; *p* = 0.034), history of PAD (OR 5.45, 95% C.I. 3.34–8.88; *p* < 0.001), and previous TIA/stroke (OR 6.34, 95% C.I. 4.12–9.76; *p* < 0.001). Similarly, at multivariable analysis, history of solid cancer (OR 12.86, 95% C.I. 7.42–22.3; *p* < 0.001) and previous DVT (OR 2.02, 95% C.I. 1.21–3.38; *p* = 0.011) were independently associated with higher incidence of venous thrombosis.

Of note, COVID-19 diagnosis was neither a predictor of arterial (*p* = 0.33) nor venous (*p* = 0.48) thrombotic complication.

#### 3.4.2. Total Thrombotic Events

Many clinical characteristics, including COVID-19 diagnosis, were significantly associated with any thrombotic event at univariate analysis (Table S1).

Clinical variables independently associated with increased rate of any thrombotic event at multivariable analysis, however, only included age (OR 1.01, 95% C.I. 1.00–1.02; *p* = 0.011), history of PAD (OR 3.33, 95% C.I. 2.14–5.20; *p* < 0.001), previous TIA/stroke (OR 3.48, 95% C.I. 2.36–5.14; *p* < 0.001), and history of DVT (OR 6.95, 95% C.I. 4.35–12.3; *p* < 0.001).

#### 3.4.3. Clinical Predictors of Thrombotic Events in COVID-19 and No-COVID-19 Groups

Table 7 shows the predictors of thrombotic events in patients of the COVID-19 and no-COVID-19 groups. Clinical variables that maintained an independent association with outcomes at multivariable analysis showed some differences between the two groups. 

Age, previous DVT, and previous TIA/stroke were independent predictors of thrombotic events both in the COVID-19 and in the no-COVID-19 groups, whereas PAD (*p* < 0.001), therapy with subcutaneous heparin (*p* = 0.025), and mild cognitive impairment (*p* = 0.005) showed an independent association with the outcome in the no-COVID-19 group only (Table 7). 

Age, PAD, and previous TIA/stroke remained associated with arterial thrombosis at multivariable analysis both in the COVID-19 and the no-COVID-19 group.

Finally, previous DVT (*p* < 0.001), cancer (*p* = 0.012), and COPD (*p* = 0.027) were independent predictors of venous thrombosis in COVID-19 patients, whereas only DVT (*p* < 0.001) and therapy with DOACs on admission (*p* = 0.038) showed an independent association with venous thrombosis in the no-COVID-19 group.

## 4. Discussion

The main data emerging from our study can be summarized as follows: (1) patients with AIRD symptoms hospitalized because of COVID-19 and treated with parenteral anticoagulant therapy did not show significantly different rates of arterial and venous thrombotic complication as compared to patients with AIRD caused by other pathogens; and (2) there were some differences in the individual clinical variables independently associated with arterial as compared with venous thrombotic events in patients with COVID-19 and other forms of AIRD, suggesting some possible different pathogenesis for arterial and venous thrombotic complications. In particular, age, diabetes mellitus, and previous history of arterial disease may predispose to arterial thrombotic events during hospitalization for AIRD whereas venous thrombosis and PE are independently associated with a history of solid tumor and previous DVT, which may be considered a predisposing factor for VTE recurrence during hospitalization. 

SARS-CoV-2 infectious disease (COVID-19) is causing a dramatic pandemia, with clusters of elevated mortality related to diffuse acute interstitial pneumonia and severe respiratory distress syndrome [1,2,3,4,5,6,7,32]. Some studies, however, have suggested that up to 40% of COVID-19 patients have an increased risk of both arterial and venous thrombotic events [9,10,11,12,13,24]. It has been suggested that the procoagulant state is mediated by the release of excessive inflammatory cytokines [33]. Alternatively, endothelial cells can be directly infected through the ACE-2 receptor, leading to platelet receptor activation and local coagulation [34,35,36,37]. Thus, due to the possible relationship between COVID-19 and hypercoagulability, and also to the obvious increased thrombotic risk in hypoxic patients, even in the absence of a high level of evidence, most centers have consequently modified their pharmacological thromboprophylaxis strategy toward therapeutic anticoagulation for routine care of COVID-19 patients [38,39,40].

However, randomized controlled trials are still on course to establish doses, duration, and real effectiveness of anticoagulant therapy for COVID-19 patients.

Some retrospective observational studies suggested that anticoagulation beyond standard prophylactic doses is associated with reduced mortality [17], but others did not confirm these findings [18]. Similarly, a small randomized trial suggested clinical benefits with therapeutic anticoagulation compared with standard prophylaxis [19], whereas the recent INSPIRATION randomized clinical trial showed that the use of intermediate-dose prophylactic anticoagulation did not result in a significant difference in the primary composite outcome of venous or arterial thrombosis, treatment with extracorporeal membrane oxygenation, or mortality, compared to standard-dose prophylactic anticoagulation [20]. A few studies have raised concern that despite anticoagulant administration, up to 30% of patients with severe COVID-19 remain at high risk of thromboembolic events [24,25], with an incidence of thrombotic complications remarkably higher compared with that of other patient categories.

Our retrospective data suggest that COVID-19 patients who receive early parenteral anticoagulant therapy show a rate of arterial and venous thrombotic complications, which is not significantly different compared with those of patients with AIRD due to other pathogens. Furthermore, although the risk of bleeding was certainly increased by pharmacological thromboprophylaxis, there were no differences in the occurrence of major, clinically relevant non-major, and minor bleeding between treated and untreated patients. Given this, our findings seem to justify and strengthen the recommendation to administer pharmacological thromboprophylaxis in hospitalized COVID-19 patients, to bring their thromboembolic risk back to the level of untreated patients with different forms of AIRD. The timing and the optimal dosing of therapeutic anticoagulation in COVID-19 patients warranted further prospective investigations. It might be useful to start preventive anticoagulation at the early stage of the disease since a large number of thromboembolic events were reported within 24 h of admission [41].

Importantly, the analysis of clinical factors independently associated with arterial and venous thromboembolic risk suggests that some subcategories of patients may benefit from earlier and stronger anticoagulation strategies. In particular, age and a previous history of either arterial, venous, or both thrombotic events (e.g., PAD, stroke, DVT) seem to be independently associated with increased risk of thrombosis in hospitalized COVID-19 patients, despite parenteral anticoagulation. Furthermore, malignancies and COPD were found to be independent predictors of venous thrombotic events in COVID-19 patients, but not in patients of the no-COVID-19 group, suggesting greater caution and earlier anticoagulation in this subgroup of patients.

Interestingly, while some clinical variables were an independent predictor of thrombotic complications both in COVID-19 and no-COVID-19 patients, we found some differences between the two groups in the individual clinical variables associated with an increased risk of arterial and venous thrombosis. Previous treatment with heparins seems to be effective in reducing the rate of thrombosis in no-COVID-19 patients, whereas it is not significantly associated with outcome at multivariate analysis in the COVID-19 group. Similarly, a known history of malignancies and COPD is associated with a higher rate of venous thrombosis in no-COVID-19 but not in COVID-19 patients. However, whether this difference between the two groups indicates a true different prognostically relevant effect of the anticoagulation strategy for COVID-19 patients or it is merely related to chance, cannot be derived from our data and, therefore, remains to be clarified. 

## 5. Limitations of the Study

Some limitations of our study should be acknowledged. First, all COVID-19 patients received parenteral heparin according to our institutional guidelines, whereas most patients with AIRD caused by other pathogens did not receive any antithrombotic therapy. Therefore, in this retrospective observational study, by design, we cannot compare treated and untreated patients and establish how effective anticoagulant therapy is in preventing arterial and venous thrombosis. Second, we did not have detailed data about pharmacological therapy, beyond heparin, administered to our patients during the hospitalization period and therefore, we could not establish how treatment influenced the occurrence of thrombotic events. Finally, since we were specifically interested in assessing whether COVID-19 showed a higher rate of thrombotic complications, we included any AIRD not attributable to SARS-CoV-2 infection in the no-COVID-19 group; therefore, we cannot establish whether differences exist in the rate of thrombosis among this heterogeneous group of patients according to the microbial agent responsible for the AIRD.

## 6. Conclusions

In conclusion, our data show that hospitalized patients with COVID-19, treated early with parenteral heparin at standard dose, do not seem to show significant differences in the cumulative incidence as well as, distinctly, in the rate of arterial and venous thrombotic events compared with AIRD patients with a different etiological diagnosis. Importantly, some clinical variables seem to be helpful to identify patients at increased risk of thrombotic complications among the whole population of AIRD patients as well as, separately, in the groups of COVID-19 and no-COVID-19 patients. 

## Figures and Tables

**Table 1:** Main clinical characteristics of the two groups of patients.

	COVID-19 Group (*n* = 2579)	no-COVID-19 Group (*n* = 2163)	*p*
Age (years)	69.0 ± 14.9	72.5 ± 15.6	<0.001
Male sex (%)	1588 (61.6)	1245 (57.6)	0.005
Hypertension (%)	1001 (38.8)	703 (32.5)	<0.001
Diabetes mellitus (%)	547 (21.2)	412 (19.0)	0.070
Obesity (%)	47 (1.8)	56 (2.6)	0.073
Charlson Comorbidity Index (n)	3.5 ± 2.2	4.6 ± 2.6	<0.001
Known ischemic heart disease (%)	306 (11.9)	300 (13.9)	0.040
Chronic heart failure (%)	190 (7.4)	386 (17.8)	<0.001
Peripheral artery disease (%)	58 (2.2)	96 (4.4)	<0.001
Previous TIA/stroke (%)	84 (3.3)	127 (5.9)	<0.001
COPD (%)	221 (8.6)	657 (30.4)	<0.001
Chronic kidney disease (%)	219 (8.5)	143 (6.6)	0.016
Chronic liver disease (%)	36 (1.4)	37 (1.7)	0.41
Solid tumor (%)	92 (3.6)	246 (11.4)	<0.001
Leukemia/Lymphoma (%)	39 (1.5)	42 (1.9)	0.26
Previous DVT (%)	22 (0.9)	56 (2.6)	<0.001
Platelet disorders (%)	19 (0.7)	21 (1.0)	0.43
Connective tissue disease (%)	19 (0.7)	40 (1.8)	0.001
Cognitive impairment (%)	173 (6.7)	428 (19.8)	<0.001
HIV infection (%)	14 (0.5)	28 (1.3)	0.008
*Pharmacological therapy on admission*			
Low-dose ASA (%)	400 (15.5)	520 (24.0)	<0.001
P2Y12 inhibitors (%)	119 (4.6)	146 (6.7)	0.001
Vitamin K antagonists (%)	56 (2.2)	66 (3.1)	0.065
DOACs (%)	150 (5.8)	174 (8.0)	0.003
Heparins (%)	346 (13.4)	139 (6.4)	<0.001
Statins (%)	166 (6.4)	214 (9.9)	<0.001

TIA = transient ischemic attack; COPD = chronic obstructive pulmonary disease; DVT = deep venous thrombosis; HIV = human immunodeficiency virus; ASA = acetylsalicylic acid; DOACs = direct oral anticoagulants.

**Table 2:** Arterial and venous thrombosis in COVID-19 and no-COVID-19 patients.

	COVID-19 Group (*n* = 2579)	no-COVID-19 Group (*n* = 2163)	*p*	*p* *
*Total thrombotic events*	121 (4.7)	150 (6.9)	<0.001	0.11
*Arterial thrombosis*	63 (2.4)	79 (3.7)	0.016	0.26
Acute myocardial infarction (%)	23 (0.9)	16 (0.7)	0.63	-
Ischemic stroke (%)	37 (1.4)	49 (2.3)	0.038	0.088
Peripheral artery thrombosis (%)	4 (0.2)	14 (0.6)	0.008	0.96
*Venous thrombosis*	61 (2.4)	71 (3.3)	0.063	0.38
Deep venous thrombosis (%)	6 (0.2)	19 (0.9)	0.002	0.028
Pulmonary embolism (%)	51 (2.0)	55 (2.5)	0.20	-
Others (%)	5 (0.2)	8 (0.4)	0.28	-

*p* * = *p* value after adjustment for clinical variables.

**Table 3:** Main clinical characteristics of propensity score-matched subgroups of patients.

	COVID-19 Group (*n* = 1518)	no-COVID-19 Group (*n* = 1518)	SMD	*p*
Age (years)	70.2 ± 16.6	69.9 ± 15.0	0.02	0.62
Male sex (%)	899 (59.2)	909 (59.9)	0.02	0.74
Hypertension (%)	478 (31.5)	484 (31.9)	0.01	0.84
Diabetes mellitus (%)	302 (19.9)	273 (18.0)	0.07	0.19
Obesity (%)	36 (2.4)	31 (2.0)	0.08	0.62
Charlson Comorbidity Index (n)	3.8 ± 2.3	3.8 ± 2.4	0.00	0.95
Known ischemic heart disease (%)	202 (13.3)	173 (11.4)	0.09	0.11
Chronic heart failure (%)	165 (10.9)	143 (9.4)	0.08	0.21
Peripheral artery disease (%)	45 (3.0)	38 (2.5)	0.08	0.50
Previous TIA/stroke (%)	71 (4.7)	67 (4.4)	0.03	0.79
COPD (%)	212 (14.0)	197 (13.0)	0.05	0.46
Chronic kidney disease (%)	96 (6.3)	105 (6.9)	0.05	0.56
Chronic liver disease (%)	30 (2.0)	27 (1.8)	0.06	0.69
Solid tumor (%)	91 (6.0)	105 (6.9)	0.08	0.30
Leukemia/Lymphoma (%)	29 (1.9)	31 (2.0)	0.04	0.90
Previous DVT (%)	22 (1.4)	26 (1.7)	0.09	0.66
Platelet disorders (%)	14 (0.9)	11 (0.7)	0.10	0.69
Connective tissue disease (%)	16 (1.1)	20 (1.3)	0.08	0.62
Cognitive impairment (%)	162 (10.7)	167 (11.0)	0.02	0.81
HIV infection (%)	13 (0.9)	15 (1.0)	0.08	0.70
*Pharmacological therapy on admission*				
Low-dose ASA (%)	322 (21.2)	317 (209)	0.01	0.86
P2Y12 inhibitors (%)	85 (5.6)	92 (6.1)	0.05	0.64
Vitamin K antagonists (%)	38 (2.5)	32 (2.1)	0.09	0.47
DOACs (%)	110 (7.2)	95 (6.3)	0.08	0.28
Heparins (%)	106 (7.0)	116 (7.6)	0.05	0.53
Statins (%)	128 (8.4)	125 (8.2)	0.01	0.90

SMD = standardized mean difference; TIA = transient ischemic attack; COPD = chronic obstructive pulmonary disease; DVT = deep venous thrombosis; HIV = human immunodeficiency virus; ASA = acetylsalicylic acid; DOACs = direct oral anticoagulants.

**Table 4:** Arterial and venous thrombosis in propensity score-matched subgroups of patients.

	COVID-19 Group (*n* = 1518)	no-COVID-19 Group (*n* = 1518)	*p*
*Total thrombotic events*	74 (4.9)	88 (5.8)	0.29
*Arterial thrombosis*	37 (2.4)	40 (2.6)	0.82
Acute myocardial infarction (%)	11 (0.7)	11 (0.7)	1.00
Ischemic stroke (%)	24 (1.6)	27 (1.8)	0.78
Peripheral artery thrombosis (%)	3 (0.2)	2 (0.1)	1.00
*Venous thrombosis*	37 (2.4)	48 (3.2)	0.27
Deep venous thrombosis (%)	4 (0.3)	12 (0.8)	0.076
Pulmonary embolism (%)	30 (2.0)	36 (2.4)	0.53
Others (%)	4 (0.3)	6 (0.4)	0.75

**Table 5:** Cumulative arterial thrombosis in the whole population.

	YES (*n* = 142)	NO (*n* = 4600)	*p* univariate	OR	95% C.I.	*p* multivariate
COVID-19 diagnosis (%)	63 (44.4)	2516 (54.7)	0.016			
Age (years)	76.8 ± 11.0	70.4 ± 15.4	<0.001	1.02	1.01–1.03	0.006
Male sex (%)	90 (63.4)	2743 (59.6)	0.37			
Hypertension (%)	58 (40.8)	1646 (35.8)	0.22			
Diabetes mellitus (%)	46 (32.4)	913 (19.8)	<0.001	1.50	1.03–2.19	0.034
Obesity (%)	5 (3.5)	98 (2.1)	0.27			
Charlson Comorbidity Index (n)	5.2 ± 2.0	3.9 ± 2.5	<0.001			
Known ischemic heart disease (%)	28 (19.7)	578 (12.6)	0.013			
Chronic heart failure (%)	21 (14.8)	555 (12.1)	0.33			
Peripheral artery disease (%)	27 (19.0)	127 (2.8)	<0.001	5.45	3.34–8.88	<0.001
Previous TIA/stroke (%)	37 (26.1)	174 (3.8)	<0.001	6.34	4.12–9.76	<0.001
COPD (%)	40 (28.2)	838 (18.2)	0.003			
Chronic kidney disease (%)	9 (6.3)	353 (7.7)	0.56			
Chronic liver disease (%)	2 (1.4)	71 (1.5)	0.90			
Solid tumor (%)	7 (4.9)	331 (7.2)	0.30			
Leukemia/Lymphoma (%)	1 (0.7)	80 (1.7)	0.36			
Previous DVT (%)	2 (1.4)	76 (1.7)	0.82			
Platelet disorders (%)	0 (0.0)	40 (0.9)	1.00			
Connective tissue disease (%)	0 (0.0)	59 (1.3)	1.00			
Cognitive impairment (%)	20 (14.1)	581 (12.6)	0.61			
HIV infection (%)	0 (0.0)	42 (0.9)	1.00			
*Pharmacological therapy on admission*						
Low-dose ASA (%)	38 (26.8)	882 (19.2)	0.025			
P2Y12 inhibitors (%)	16 (11.3)	246 (5.4)	0.004			
Vitamin K antagonists (%)	6 (4.2)	116 (2.5)	0.21			
DOACs (%)	16 (11.3)	308 (6.7)	0.036			
Heparins (%)	11 (7.7)	474 (10.3)	0.32			
Statins (%)	18 (12.7)	362 (7.9)	0.040			

OR = odds ratio; TIA = transient ischemic attack; COPD = chronic obstructive pulmonary disease; DVT = deep venous thrombosis; HIV = human immunodeficiency virus; ASA = acetylsalicylic acid; DOACs = direct oral anticoagulants.

**Table 6:** Cumulative venous thrombosis in the whole population.

	YES (*n* = 132)	NO (*n* = 4610)	*p* univariate	OR	95% C.I.	*p* multivariate
COVID-19 diagnosis (%)	61 (46.2)	2518 (54.6)	0.057			
Age (years)	71.8 ± 13.6	70.5 ± 15.3	0.37			
Male sex (%)	86 (65.2)	2747 (59.6)	0.20			
Hypertension (%)	50 (37.9)	1654 (35.9)	0.64			
Diabetes mellitus (%)	21 (15.9)	938 (20.3)	0.21			
Obesity (%)	2 (1.5)	101 (2.2)	0.60			
Charlson Comorbidity Index (*n*)	4.2 ± 2.4	4.0 ± 2.5	0.23			
Known ischemic heart disease (%)	15 (11.4)	591 (12.8)	0.62			
Chronic heart failure (%)	13 (9.8)	563 (12.2)	0.41			
Peripheral artery disease (%)	2 (1.5)	152 (3.3)	0.27			
Previous TIA/stroke (%)	2 (1.5)	209 (4.5)	0.11			
COPD (%)	26 (19.7)	852 (18.5)	0.72			
Chronic kidney disease (%)	7 (5.3)	355 (7.7)	0.31			
Chronic liver disease (%)	3 (2.3)	70 (1.5)	0.49			
Solid tumor (%)	20 (15.2)	318 (6.9)	<0.001	12.86	7.42–22.3	<0.001
Leukemia/Lymphoma (%)	1 (0.8)	80 (1.7)	0.41			
Previous DVT (%)	20 (15.2)	58 (1.3)	<0.001	2.02	1.21–3.38	0.007
Platelet disorders (%)	3 (2.3)	37 (0.8)	0.082			
Connective tissue disease (%)	1 (0.8)	58 (1.3)	0.61			
Cognitive impairment (%)	12 (9.1)	589 (12.8)	0.21			
HIV infection (%)	1 (0.8)	41 (0.9)	0.87			
*Pharmacological therapy on admission*						
Low-dose ASA (%)	22 (16.7)	898 (19.5)	0.42			
P2Y12 inhibitors (%)	8 (6.1)	257 (5.6)	0.81			
Vitamin K antagonists (%)	0 (0.0)	122 (2.6)	1.00			
DOACs (%)	5 (3.8)	318 (6.9)	0.17			
Heparins (%)	11 (8.3)	474 (10.3)	0.47			
Statins (%)	6 (4.5)	374 (8.1)	0.14			

OR = odds ratio; TIA = transient ischemic attack; COPD = chronic obstructive pulmonary disease; DVT = deep venous thrombosis; HIV = human immunodeficiency virus; ASA = acetylsalicylic acid; DOACs = direct oral anticoagulants.

**Table 7:** Main results of multivariable analysis in the two groups of patients.

	COVID-19			no-COVID-19		
	Odds Ratio	95% C.I.	*p*	Odds Ratio	95% C.I.	*p*
*Total thrombotic events*						
Age	1.01	1.00–1.03	0.030	1.01	1.00–1.03	0.029
Previous DVT	4.53	1.48–13.8	0.008	9.98	5.27–18.9	<0.001
Previous TIA/stroke	4.36	2.39–7.95	<0.001	3.28	1.96–5.51	<0.001
Peripheral artery disease	-	-	-	4.55	2.66–7.78	<0.001
Heparin	-	-	-	0.38	0.16–0.89	0.025
Mild cognitive impairment	-	-	-	0.49	0.30–0.80	0.005
*Arterial thrombosis*						
Age	1.02	1.00–1.04	0.050	1.03	1.01–1.05	0.012
Peripheral artery disease	3.22	1.25–8.30	0.016	8.28	4.57–15.0	<0.001
Previous TIA/stroke	7.06	3.58–13.9	<0.001	6.10	3.47–10.7	<0.001
Mild cognitive impairment	-	-	-	0.51	0.27–0.96	0.038
*Venous thrombosis*						
Previous DVT	8.82	2.84–27.4	<0.001	16.63	8.66–32.0	<0.001
Solid tumor	3.11	1.28–7.55	0.012	-	-	-
COPD	2.21	1.09–4.45	0.027	-	-	-
DOACs	-	-	-	0.12	0.02–0.88	0.038

C.I.= confidence interval; DVT = deep venous thrombosis; TIA = transient ischemic attack; COPD = chronic obstructive pulmonary disease; DOACs = direct oral anticoagulants.

## Data Availability

Data available on request due to restrictions.

## Supplementary Materials

The following are available online at https://www.mdpi.com/article/10.3390/jcm10214973/s1, Table S1. Total thrombotic events in the whole population.
