# Supplementary material for: Incidence and Predictors of Thrombotic Complications in 4742 Patients with COVID-19 or Other Acute Infectious Respiratory Diseases: A Propensity Score-Matched Study
Source: J Clin Med. 2021 Oct 26;10(21):4973. doi: 10.3390/jcm10214973 (PMC8584832; doi:10.3390/jcm10214973)
Supplement: Supplementary file 1 [file jcm-10-04973-s001.zip › jcm-1433929-supplementary.pdf]

Table S1. Total thrombotic events in the whole population.

	YES (n=271)	NO (n=4471)	p univariate	OR	95% C.I.	p multivariate
COVID-19 diagnosis (%)	121 (44.6)	2458 (55.0)	0.001			
Age (years)	74.3±12.6	70.4±15.4	<0.001	1.01	1.00-1.02	0.011
Male sex (%)	173 (63.8)	2660 (59.5)	0.16			
Hypertension (%)	107 (39.5)	1597 (35.7)	0.21			
Diabetes mellitus (%)	67 (24.7)	892 (20.0)	0.058			
Obesity (%)	7 (2.6)	96 (2.1)	0.63			
Charlson Comorbidity Index (n)	4.7±2.3	3.9±2.5	<0.001			
Known ischemic heart disease (%)	43 (15.9)	563 (12.6)	0.12			
Chronic heart failure (%)	34 (12.5)	542 (12.1)	0.84			
Peripheral artery disease (%)	29 (10.7)	125 (2.8)	<0.001	3.33	2.14-5.20	<0.001
Previous TIA/stroke (%)	39 (14.4)	172 (3.8)	<0.001	3.48	2.36-5.14	<0.001
COPD (%)	66 (24.4)	812 (18.2)	0.011			
Chronic kidney disease (%)	16 (5.9)	346 (7.7)	0.27			
Chronic liver disease (%)	5 (1.8)	68 (1.5)	0.67			
Solid tumor (%)	27 (10.0)	311 (7.0)	0.063			
Leukemia/Lymphoma (%)	2 (0.7)	79 (1.8)	0.22			
Previous DVT (%)	22 (8.1)	56 (1.3)	<0.001	7.32	4.35-12.3	<0.001
Platelet disorders (%)	3 (1.1)	37 (0.8)	0.63			
Connective tissue disease (%)	1 (0.4)	58 (1.3)	0.21			
Cognitive impairment (%)	32 (11.8)	569 (12.7)	0.66			
HIV infection (%)	1 (0.4)	41 (0.9)	0.37			
Pharmacological therapy on admission						
Low-dose ASA (%)	60 (22.1)	860 (19.2)	0.24			
P2Y12 inhibitors (%)	24 (8.9)	241 (5.4)	0.017			
Vitamin K antagonists (%)	6 (2.2)	116 (2.6)	0.70			
DOACs (%)	20 (7.4)	304 (6.8)	0.71			
Heparins (%)	22 (8.1)	463 (10.4)	0.24			
Statins (%)	24 (8.9)	356 (8.0)	0.60			

OR=odds ratio; TIA=transient ischemic attack; COPD=chronic obstructive pulmonary disease; DVT=deep venous thrombosis; HIV=human immunodeficiency virus; ASA=acetylsalicylic acid; DOACs=direct oral anticoagulants.
